# Curative resection by splenectomy for solitary splenic metastasis from early gastric cancer: a case report and literature review

**DOI:** 10.1186/s12885-017-3434-y

**Published:** 2017-06-20

**Authors:** Junichi Yoshizawa, Naoki Kubo, Satoshi Ishizone, Fumitoshi Karasawa, Ataru Nakayama

**Affiliations:** 1 Department of Surgery, North Alps Medical Center Azumi Hospital, 3207-1 Ikeda, Ikeda-machi, Kitaazumi-gun, Nagano, Prefecture 399-8695 Japan; 2 Department of Surgery, Ina Central Hospital, 1313-1 Koshirokubo, Ina-shi, Nagano, Prefecture 396-8555 Japan; 3 Present address: Suwa Red Cross Hospital, 5-11-50 Kogandori, Suwa-shi, Nagano, Prefecture 392-8510 Japan

**Keywords:** Case report, Early gastric cancer, Gastric cancer, Splenectomy, Splenic metastasis

## Abstract

**Background:**

Solitary metastasis of a malignancy to the spleen is rare, particularly for gastric cancer. Only a few case reports have documented isolated splenic metastasis from early gastric cancer. We describe a case of splenic metastasis from early gastric cancer.

**Case presentation:**

A 60-year-old man underwent a distal gastrectomy for early gastric cancer. It infiltrated the submucosa with pathological nodal involvement (pT1bN2M0, stage IIB). One year after the gastrectomy, an abdominal computed tomography scan showed a low-density lesion, 17 mm in diameter, at the upper pole of the spleen. Positron emission tomography/computed tomography showed focal accumulation of fluorine-18 fluorodeoxyglucose in the spleen without extrasplenic tumor dissemination or metastasis. We diagnosed splenic metastasis of gastric cancer, and performed a splenectomy. Histological examination confirmed moderately differentiated tubular adenocarcinoma and poorly differentiated adenocarcinoma (solid type) that was consistent with the features of the primary gastric cancer. The splenic tumor was pathologically and immunohistochemically diagnosed as a metastasis from the gastric carcinoma. More than 18 months after the splenectomy, the patient has had no evidence of recurrent gastric cancer.

**Conclusion:**

When solitary metastasis to the spleen is suspected during the postoperative follow-up of a patient with gastric cancer, a splenectomy is a potentially effective treatment.

## Background

Isolated metastasis to the spleen from early gastric cancer is very rare. Once splenic metastasis from gastric cancer occurs, it is usually accompanied by multiorgan metastasis and dissemination [1–3]. Only a few case reports have documented isolated splenic metastasis from early gastric cancer [4, 5]. In this paper, we present a very rare case of a solitary splenic metastasis from early gastric cancer. The metastasis occurred 1 year after gastrectomy, and a splenectomy resulted in a curative resection. From the literature, we reviewed 19 patients who received a curative splenectomy for isolated metastasis from gastric cancer.

**Table 1:** Summary of patients of radical treatment by splenectomy for isolated splenic metastasis from gastric cancer

					Primary gastric cancer				Splenic metastasis			Prognosis	
Case	Year	Author	Age	sex	Location	Histological type	TNM	DFI	Size (mm)	Operation	Number ofmetastasis	Follow up time (mo)	Outcome
1	1983	Takebayashi [9]	28	F	U, M	Por	T3N1M1	same	NA	TG+PS	1	3	dead
2	1989	Ikeda [10]	57	M	U	Por	T3N1M0	17mo	20×15	TG→S	1	15	RFS
3	1992	Shirai [4]	63	M	M	Pap	T1N0M0	20 mo	50×45	DG→S	1	20	RFS
4	1993	Mori [11]	49	M	R	Tub	T3N3M1	same	110×65	TG+PS	1	12	RFS
5	1997	Tatsuzawa [12]	54	M	M	Tub	T2N2M0	102mo	35×25	DG→S	1	5	alive
6	1998	Sakamoto [13]	67	M	U, M	Tub	T4N2M1	same	90×35	TG+PS	1	8	dead
7	1999	Takahashi [14]	64	M	L	Tub	T2N2M0	16mo	65×55	DG→S	4	7	dead
8	2000	Opocher [15]	76	M	L	Tub	T2N0M0	57mo	80	DG→S	1	13	RFS
9			66	M	NA	Por	T2N1M0	36mo	40	TG→S	1	14	RFS
10	2002	Yamanouchi [16]	69	M	L	Tub	T2N1M0	50mo	45×40	DG→S	1	40	dead
11	2009	Sunitsch [17]	80	F	L, R	Tub	T2N0M0	37mo	150	TG→S	1	NA	NA
12	2010	Takenaga [18]	65	M	U	por	TXN1M0	41mo	38	TG→S	1	12	RFS
13	2010	Lu [19]	59	M	U	Hepatoid AC	TXNXM1	same	40	TG+S	1	18	alive
14	2010	Kawasaki [5]	76	M	U	Pap	T1N1M0	12mo	70	PG→S	1	24	RFS
15	2013	Kamaleshwaran [20]	55	M	U	NA	NA	12mo	NA	PG→S	1	NA	NA
16	2013	Zhu [21]	62	M	M, L	Por	T3N2M0	2mo	45×40	TG→S	2	9	RFS
17	2014	Kano [22]	56	F	U	AC	T4N2M1	same	NA	TG+S	1	30	RFS
18	2015	Santos [23]	71	M	NA	NA	T3N0M0	8year	25×15×10	TG→S	3	7	RFS
19		Our case	60	M	M	Tub, Por	T1N2M0	12mo	20×18	DG→S	1	18	RFS

*DFI* disease free interval, *U* the upper third of stomach, *M* middle third of stomach. *L* lower third of stomach, *R* residual stomach *Por* poorly differentiated adenocarcinoma, *Tub* tubular adenocarcinoma, *Pap* papillary adenocarcinoma, *AC* adenocarcinoma, *NA* not available, *mo* month, *TG* total gastrectomy, *DG* distal gastrectomy, *PS* pancreatosplenectomy, *S* splenectomy, *RFS* relapse free survival

## Case presentation

**Fig. 1:**
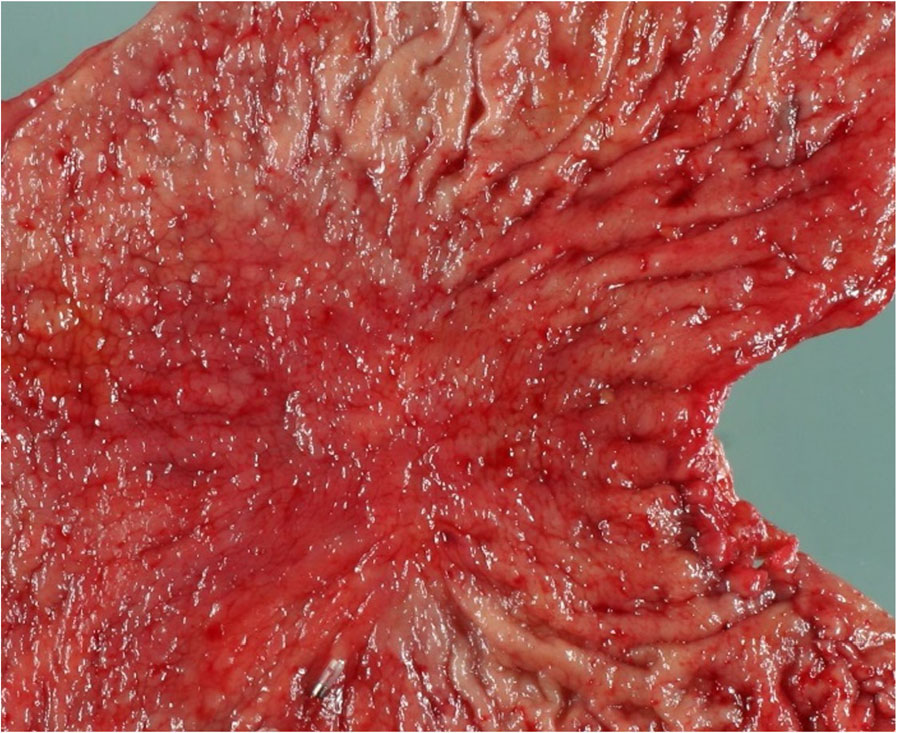
The resected specimen of stomach has a tumor 25 × 20 mm in diameter, with a depressed and elevated form (type IIa/IIc)

A 60-year-old man visited our institution because of dysphagia. He was diagnosed with early gastric carcinoma in the middle third of the stomach, based on upper gastrointestinal endoscopy and computed tomography (CT) imaging. The preoperative carcinoembryonic antigen (CEA) and carbohydrate antigen 19–9 (CA19–9) values were within the normal ranges. He underwent a distal gastrectomy with a D1+ lymph node dissection. Pathologic histology of the resected stomach macroscopically revealed a tumor, 25 × 20 mm in diameter, with a depressed and elevated form (Type IIa/IIc) (Fig. 1). A diagnosis of moderately differentiated tubular adenocarcinoma was confirmed. Poorly differentiated adenocarcinoma (solid type) infiltrated the submucosa with nodal involvement (4 of 63 nodes were positive for metastases) but without venous invasion; there was pathologically moderate lymphatic invasion. The gastric cancer fulfilled the criteria of T1bN2M0, stage IIB, based on the American Joint Committee on Cancer TNM staging classification for carcinoma of the stomach (7th edition, 2012) [6]. The patient received one cycle of oral chemotherapy consisting of S-1; however, treatment was discontinued because of the adverse events of nausea, loss of appetite, and loss of body weight.

**Fig. 2:**
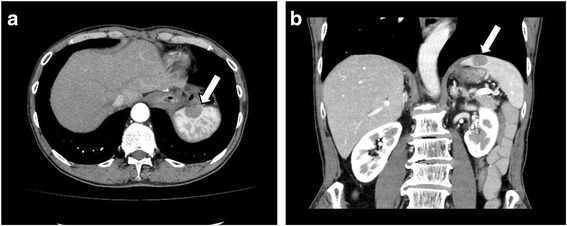
The CT scan reveals a low-density lesion that is 17 mm in diameter and at the upper pole of the spleen (*arrow*). **a**: The horizontal section. **b**: The coronal section

**Fig. 3:**
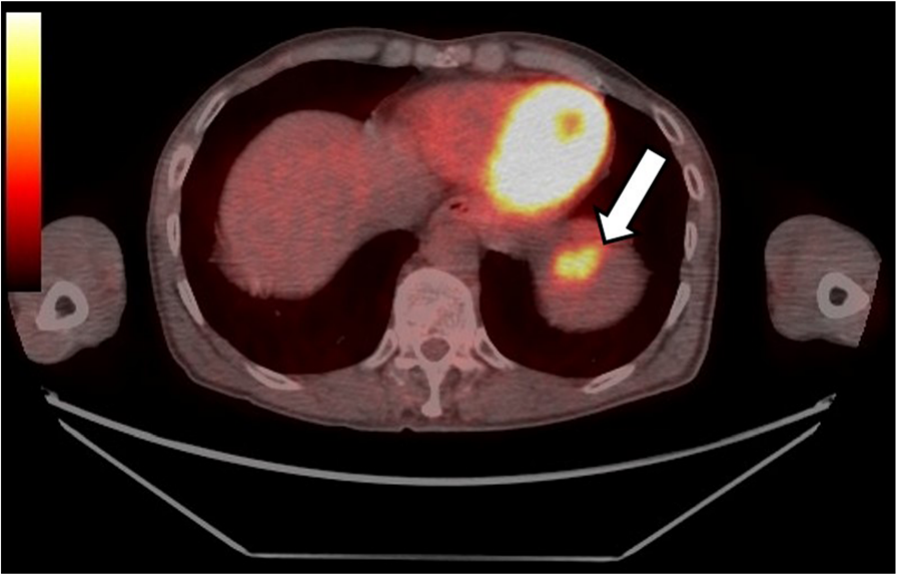
The PET-CT image shows intense fluorine-18 fluorodeoxyglucose (18F–FDG) uptake with a maximum standardized uptake value (SUV) of 9.0 (*arrow*). There is no suspected extrasplenic tumor dissemination or metastasis

Twelve months after the surgery, an abdominal CT scan showed a low-density lesion, 17 mm in diameter, at the upper pole of the spleen (Fig. 2). Whole body fluorine-18 fluorodeoxyglucose (18F–FDG) positron emission tomography/computed tomography (PET/CT) showed a hypodense mass in the spleen and intense 18F–FDG uptake with a maximum standardized uptake value (SUV) of 9.0. Extrasplenic tumor dissemination or metastasis was not suspected (Fig. 3).

Six weeks later, a follow-up CT scan revealed enlargement of the splenic lesions (22 × 17 mm) with obvious contrast enhancement and no evidence of other metastasis. Upper gastrointestinal endoscopy findings were negative in the residual stomach. Colonoscopy revealed no abnormalities. During the postoperative follow-up, the serum CEA and CA19–9 levels were within normal limits.

We finally suspected that the tumor was a solitary splenic metastasis of the gastric cancer. The patient underwent a laparotomy because his splenic metastasis was isolated and resectable. In addition, there were no other metastases.

The laparotomy revealed no lymph node involvement, hepatic metastasis, or peritoneal dissemination. The tumor was in the upper pole of the spleen. The splenectomy preserved the residual stomach. The patient’s postoperative period was uneventful.

**Fig. 4:**
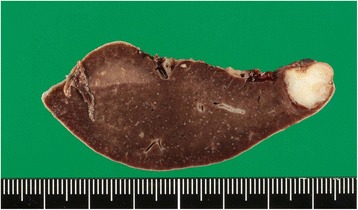
The specimen is a white mass without a capsule and measures 20 × 18 mm. It is at the upper pole of the spleen

**Fig. 5:**
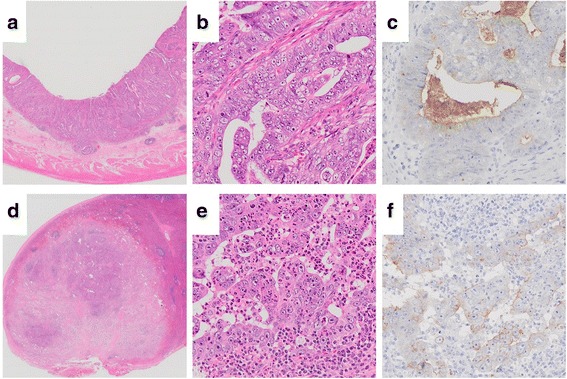
The microscopic findings of the gastric cancer (**a**-**c**) and the splenic tumor (**d**-**e**). **a**: The gastric cancer has infiltrated the submucosa (H-E; magnification, ×40). **b**: The moderately differentiated tubular adenocarcinoma and poorly differentiated adenocarcinoma (solid type) are confirmed in the gastric specimen (H-E, magnification, ×200). d and e: The histological examination reveals a moderately differentiated tubular adenocarcinoma and poorly differentiated adenocarcinoma (solid type) in the splenic tumor (**d**: H-E; magnification, ×40; **e**: H-E; magnification, ×200). **c** and **f**: The immunohistochemical expression of CEA is positive both in the primary gastric cancer and in the splenic tumor (CEA; magnification, ×200). CEA, carcinoembryonic antigen; H-E, hematoxylin-eosin stain

The specimen was a white mass without a capsule that measured 20 × 18 mm; it was at the upper pole of the spleen (Fig. 4). Histological examination revealed a moderately differentiated tubular adenocarcinoma and poorly differentiated adenocarcinoma (solid type). These features were similar to those of the primary gastric cancer. The immunohistochemical expression of CEA was positive both in the primary gastric cancer and in the splenic tumor (Fig. 5). These histological and immunohistochemical profile findings were consistent with the metastasis of a splenic tumor from the primary gastric cancer.

The patient refused to receive adjuvant chemotherapy. For 18 months, he has been well and healthy without any evidence of gastric cancer recurrence.

## Discussion

The spleen is a hypervascular organ; however, splenic malignancy or metastasis is rare, except in cases of malignant lymphoma and leukemia [7]. The frequency of splenic metastasis to many visceral organs at the terminal stage of cancer is high. Berge et al. reviewed 4400 autopsy cases associated with metastatic cancer, and reported 312 (7.1%) cases of splenic metastasis; of these, 22 cases resulted from gastric cancer (4.1%; 533 gastric cancer cases) [1]. Lam et al. reviewed 12,399 autopsy cases, which included uncomplicated malignant tumors, and reported 92 cases of splenic metastasis in which the primary tumors were breast cancer (22.9%), lung cancer (20.2%), colorectal cancer (9.4%), ovarian cancer (9%), and gastric cancer (6.9%) [2]. Splenic metastasis is divided into 2 categories: synchronous metastasis and metachronous metastasis. The time interval from the occurrence of the primary tumor to metachronous splenic metastasis varies by the kind of malignancy.

By contrast, isolated splenic metastasis of early stage cancer is rare. Although it is not clear why splenic metastasis rarely occurs from malignancies, several hypotheses have been proposed [2, 7]. First, the spleen has a poorly developed lymph system, particularly for afferent lymphatics. Metastasis to the spleen via the lymphatic system is consequently rare. Second, the splenic artery, as a main afferent vessel to the spleen, divaricates sharply from the celiac trunk; as a result, tumor cells have difficulty passing through to the spleen. Third, the spleen constricts rhythmically, so that tumor cells tend to be squeezed out of it. Fourth, as an endothelial system organ, the spleen has an antitumor effect and therefore provides an immunologically unfavorable environment for malignant cells to grow.

Splenic metastases from gastric cancer are rare, regardless of their proximity anatomically. Compét et al. reviewed 93 cases of isolated splenic metastasis, and reported that the primary sites of malignancies were the colorectum (20 cases), ovary (18 cases), lung (10 cases), endometrium (9 cases), kidney (9 cases), and stomach (7 cases) [8].

For the present report, searches were performed for related reports published between April 1983 and October 2016 in Japan Medical Abstracts Society Web (the largest medical database in Japanese), and from the earliest possible date to October 2016 in PubMed, using the keywords “splenic metastasis,” “gastric cancer,” and “gastric neoplasms”. To our knowledge, there have been 19 reported cases of radical treatment by splenectomy for isolated splenic metastasis from gastric cancer, which comprised 5 cases of synchronous metastasis and 14 cases of metachronous metastasis (including our case) (Table 1) [4, 5, 9–23]. In our analysis of these cases, categorization by sex revealed a male predominance (16 men and 3 women). Categorization by age revealed that 11 of 19 patients were younger than 65 years and that the mean age was 61.9 years (range, 28–80 years), indicating a predominance in the younger population. The histological type varied and included papillary adenocarcinoma, tubular adenocarcinoma, poorly differentiated adenocarcinoma, and hepatocellular adenocarcinoma. The invasion depth of the primary gastric cancer ranged from T1 to T4. In particular, only 3 cases (including our case) were early gastric cancer, which was confined to the mucosa or submucosa, regardless of lymph node metastasis. Thirteen of 18 patients had lymph node metastasis. All synchronous splenic metastases were accompanied by residual gastric cancer or involved the upper third of the stomach, and the patients received total gastrectomy and splenectomy. Metachronous splenic metastasis developed from various sites in the stomach, and the period from gastric resection to splenic metastasis varied from 2 months to 8 years.

A gastric cancer can result in splenic metastasis by 3 pathways: (1) via the splenic artery, (2) via the splenic vein, and (3) via the lymphatic route [24]. For the splenic vein route, the tumor cells usually need to flow retrogradely through the splenic vein. Therefore, metastasis would be uncommon, unless the patient has hepatic disease with portal hypertension or thrombosis of the splenic vein. For the metastatic route by the splenic artery, tumor cells flow into the spleen via systemic circulation. Therefore, splenic metastasis usually occurs as a multivisceral organ metastasis, and rarely occurs as an isolated metastasis only to the spleen [16].

In our patient, the route of metastasis to the spleen was unknown. However, we found lymph node metastases during the gastrectomy (N2). Vascular invasion in the cancer specimen was not histologically identified. In addition, the gastric cancer was early stage cancer with submucosal invasion and without the indications for liver disease. We accordingly considered the possibility that isolated splenic metastasis developed via the lymphatic route.

Most isolated splenic metastases are asymptomatic. However, isolated splenic metastasis is sometimes detected when examining a patient for general fatigue, weight loss, abdominal pain, splenomegaly, anemia, or thrombocytopenia [8]. Isolated splenic metastasis is usually diagnosed on investigating a primary cancer, or on follow-up postoperative ultrasonography or CT scanning. On a CT scan, splenic metastases often appear as cystic degeneration, a solid tumor, or a calcified tumor. Hence, it was not necessarily easy to distinguish splenic metastasis from a splenic benign tumor or lymphoma [21, 25].

Cavanna et al. reported 160 cases of splenic tumor for which the patients underwent fine needle aspiration (FNA) for diagnosis [26]. They described an accuracy rate of 98.1% without complications, and concluded that FNA for splenic tumor is safe and valid. However, it is generally avoided because of the risk of bleeding from the spleen or intra-abdominal dissemination of the tumor.

A PET/CT scan is useful for differentiating between malignant and benign tumors, and for assessing metastasis to many organs or para-aortic lymph nodes [20]. In the present case, PET/CT revealed limited abnormal accumulation of 18F–FDG to the spleen; based on this finding, we suspected isolated splenic metastasis from gastric cancer. We finally diagnosed the tumor, based on pathologic findings that were very similar to the histologic form and immunostaining findings similar to the primary gastric cancer.

Splenectomy provides the only reliable possibility for curative treatment of solitary splenic metastasis from gastric cancer [8]. The aims of splenectomy are to remove grossly visible tumor tissue to the maximum extent possible and to obtain histologically free surgical margins. However, even if an isolated splenic tumor is observed and identified as a suspected metastasis in the course of follow-up for gastric cancer, it may only be the initial finding of a systemic visceral metastasis because the splenic lesion is usually accompanied by multiple metastases at various other sites [1–3]. In this situation, if splenectomy were performed, the patient would subsequently have a relapse in another organ. Furthermore, surgical stress could have an adverse effect on the patient. With this in mind, the effectiveness of a wait-and-see approach was suggested in the past literature for cases of suspected solitary splenic metastases from gastric cancer [5, 21, 23]. The wait-and-see approach means that the patient is followed-up using imaging tests for a short interval of time, instead of undergoing splenectomy immediately after the discovery of solitary splenic metastasis from gastric cancer. This approach is considered to be useful for detecting cases with occult metastases from gastric cancer other than splenic metastasis. By using a wait-and-see approach, we may be able to select patients who would obtain true benefits from splenectomy. However, there is no established consensus regarding the interval that should be used for imaging assessments under the wait-and-see approach. In previously published studies, imaging tests were performed using CT scans in a short period of 1–2 months to 4 months, during which time chemotherapy was also administered. Therefore, we waited and observed the patient for 6 weeks, during which chemotherapy was not provided because the patient declined it. The second CT scan did not reveal findings of other metastases, whereas the splenic metastasis grew in size. After the second CT scan, we performed splenectomy, because we feared progression in the form of dissemination or invasion from splenic metastasis, which could have exposed the outside of the spleen if we had waited for another follow-up interval. As stated above, it may be beneficial to use a wait-and-see approach to assess the presence of splenic and systemic visceral metastases, and thereby determine whether to perform splenectomy for solitary splenic metastasis from gastric cancer.

The prognosis of an isolated splenic metastasis from gastric cancer treated by splenectomy is unclear because of its rarity. All past reports [4, 5, 9–23] have been case reports only, and the postsurgical follow-up periods were comparatively short. One study reported that it is possible for some patients with synchronous splenic metastasis to obtain long-term survival, whereas patients with metachronous metastasis have a poor prognosis [21]. In our investigation, 2 of 5 cases of fatal synchronous splenic metastasis and 2 of 14 cases of metachronous metastasis from gastric cancer were treated by splenectomy (Table 1). In addition, among the 14 successfully treated metachronous metastasis cases, 8 cases demonstrated relapse-free survival for longer than 12 months. Hence, the prognosis of isolated metachronous splenic metastasis from gastric cancer may be favorable. However, more investigations and longer follow-up durations are necessary.

Overwhelming postsplenectomy infection (OPSI) is a possible critical life-threatening complication. An OPSI can result in sepsis and meningitis after splenectomy, and 3.2% of patients who have undergone a splenectomy acquire an OPSI. The risk of OPSI continues throughout a patient’s life after splenectomy. The most frequent causative agent of an OPSI is *Streptococcus pneumoniae*, followed by *Haemophilus influenzae* type b and *Neisseria meningitides*. Preventing OPSI involves administering a pneumococcal polysaccharide vaccine and immunizing a patient against *H. influenzae* type b and *N. meningitides*. The incidence of OPSI is infrequent; however, once it occurs, the mortality rate is very high, at 50%–70% [27]. Preventing OPSI is very important, and efforts are needed to educate healthcare workers and patients about this complication.

## Conclusions

We presented a rare case of solitary splenic metastasis after curative resection of early gastric cancer. The metastasis was treated by splenectomy. When a solitary mass in the spleen is detected at the diagnosis of gastric cancer or during the postoperative follow-up of a patient with gastric cancer, even early stage gastric cancer, the mass may be a splenic metastasis. PET/CT scanning is useful for diagnosing splenic metastasis and for assessing metastasis to other organs or the para-aortic lymph nodes. If a solitary metastasis to the spleen without distant metastasis is suspected, a splenectomy should be considered for a curative treatment.

## Abbreviations

18F–FDG: Fluorine-18 fluorodeoxyglucose
CA 19–9: Carbohydrate antigen 19–9
CEA: Carcinoembryonic antigen
CT: Computed tomography
FNA: Fine needle aspiration
H-E: Hematoxylin-eosin stain
OPSI: Overwhelming postsplenectomy infection
PET: Positron emission tomography
SUV: Standardized uptake value.
